# Dual‐center randomized clinical trial exploring the optimal duration of antimicrobial prophylaxis in patients undergoing pancreaticoduodenectomy following biliary drainage

**DOI:** 10.1002/ags3.12209

**Published:** 2018-09-17

**Authors:** Tomohisa Yamamoto, Sohei Satoi, Tsutomu Fujii, Suguru Yamada, Hiroaki Yanagimoto, So Yamaki, Hideki Takami, Satoshi Hirooka, Hisashi Kosaka, Masaya Kotsuka, Takayuki Miyara, Yasuhiro Kodera

**Affiliations:** ^1^ Department of Surgery Kansai Medical University Hirakata Japan; ^2^ Department of Surgery and Science Graduate School of Medicine and Pharmaceutical Sciences for Research University of Toyama Toyama Japan; ^3^ Department of Gastroenterological Surgery (Surgery II) Nagoya University Graduate School of Medicine Nagoya Japan; ^4^ First Department of Internal Medicine Kansai Medical University Hirakata Japan

**Keywords:** antimicrobial prophylaxis, cefozopran, infectious complication, pancreaticoduodenectomy, randomized controlled study

## Abstract

**Objectives:**

The aim of this dual‐center randomized controlled trial was to determine the optimal duration of antimicrobial prophylaxis in patients treated with pancreaticoduodenectomy (PD) who underwent preoperative biliary drainage (PBD) but were without cholangitis.

**Background:**

Some reports showed that PBD in patients undergoing pancreatectomy increased the rate of perioperative complications. However, no clinical trial has evaluated the optimal duration of antimicrobial prophylaxis with a focus on patients who underwent PD following PBD.

**Methods:**

A total of 82 patients who underwent PD between March 2012 and December 2016 were randomly assigned to either a 1‐day group (n = 40), in which cefozopran (CZOP) as antimicrobial prophylaxis was given only on the day of surgery, or a 5‐day group (n = 42), in which CZOP was given for 5 consecutive days beginning on the day of surgery. We evaluated the incidence of infectious and other complications after PD.

**Results:**

Outcomes were significantly better in the 1‐day group compared with the 5‐day group (*P* < 0.05) in terms of the incidence of overall infectious complications (15% vs 36%, respectively), intra‐abdominal abscess (3% vs 21%, respectively), clinically relevant postoperative pancreatic fistula (8% vs 24%, respectively), and Clavien‐Dindo grade III‐V complications (10% vs 31%, respectively). Duration of postoperative hospital stay was significantly shorter in the 1‐day group (10 days vs 15 days, *P* = 0.018). Anaerobic bacteria and methicillin‐resistant cocci were isolated from the drainage fluid only among patients in the 5‐day group.

**Conclusion:**

Single‐day prophylactic use of CZOP is appropriate for patients who undergo PD following PBD without preoperative cholangitis.

## 1 INTRODUCTION

Pancreaticoduodenectomy (PD) is a complex surgical procedure carried out for the treatment of periampullary diseases. Although the perioperative mortality rate of PD has remained relatively low, at approximately 1%‐2% in high‐volume centers, high morbidity of roughly 50% remains a problem.^1^, ^2^, ^3^

Obstructive jaundice is the most common symptom in patients with periampullary diseases. Preoperative biliary drainage (PBD) is widely carried out for relieving biliary obstruction, but it is known to cause bile contamination in approximately 80% of patients.^4^, ^5^ However, PBD is still considered indispensable before major hepatobiliary and pancreatic surgeries, as several previous retrospective studies have reported that it reduced morbidity and mortality after PD through improving liver function.^6^, ^7^

In contrast, a randomized controlled trial (RCT) concluded that routine PBD in patients undergoing pancreatectomy for pancreatic head tumors significantly increased the rate of perioperative complications.^8^ First‐ or second‐generation cephem has been used commonly as prophylactic antibiotic in our institutes as well as in Western countries.^9^, ^10^ Furthermore, several guidelines recommend discontinuation of antimicrobial prophylaxis within 24 hours of surgery, even for major hepato‐pancreato‐biliary surgery.^11^, ^12^ In contrast, Sudo et al^4^ suggested that patients with PBD are at high risk for postoperative infectious complications, and that therapeutic antimicrobial treatment with third‐ or fourth‐generation cephalosporins targeting Gram‐negative bacilli for 3 or 4 days rather than routine antimicrobial prophylaxis is more appropriate for patients who have undergone PBD. Additionally, Sourrouille et al^13^ have reported that 5 days of postoperative antimicrobial therapy in patients at high risk of contamination reduces the overall rate of infectious complications after PD. Among patients who underwent PBD, those who had developed cholangitis defined by the Tokyo Guidelines 2007^14^ preoperatively had a particularly high likelihood of developing postoperative infectious complications, and routine use of broad‐spectrum antibiotics as therapeutic antimicrobial therapy has been implicated.^15^, ^16^ Thus, hard evidence as to the duration and selection of antibiotics for use as antimicrobial prophylaxis has been lacking for patients with an intermediate risk for infection, such as those who undergo PD after receiving PBD but have no signs of cholangitis preoperatively. In the current study, cefozopran (CZOP) which covers the *Enterococcus* and *Enterobacter* species was chosen for controlling infection in patients with PBD.

We herein report results from a RCT comparing antimicrobial prophylaxis with 1‐day versus 5‐day administration of CZOP, which is a fourth‐generation cephalosporin, in patients without cholangitis who underwent PD after PBD, with the incidence of postoperative infectious complications as a primary endpoint.

## 2 METHODS

### 2.1 Patients

**Figure 1:**
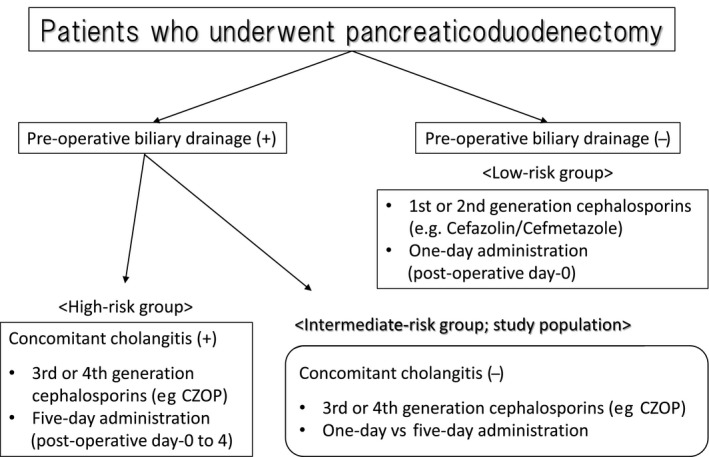
Recommended antibiotic prophylaxis according to preoperative biliary drainage. CZOP, cefozopran; CEZ, Cefazolin; CMZ, Cefmetazole

Patients with periampullary disease who were 20 years or older and who underwent PBD followed by PD at Kansai Medical University Hospital and Nagoya University Hospital were eligible for this RCT. We classified the patients who underwent PD into three risk categories according to biliary contamination or infection, as shown in Figure 1; (i) the low‐risk group: those who received no PBD preoperatively; (ii) the intermediate‐risk group: those who received preoperative PBD but were without signs of cholangitis; and (iii) the high‐risk group: those who had cholangitis and received preoperative PBD. Only patients who belonged to the intermediate‐risk group were included. Patients who were classified as high risk were excluded and routinely received antimicrobial prophylaxis with CZOP until postoperative day 4.^15^, ^16^ In this study, we always clamped the hepatic duct by clamp forceps to prevent spilling bile juice after resecting the common bile duct. Peritoneal lavage was routinely conducted using 5000 mL each of normal saline at the time when the pancreas head was removed and at the end of the operation, respectively. All patients provided written informed consent before study enrolment. This study was registered in UMIN‐CTR (UMIN000007277) and was approved by the ethics committee of each institution (H110276: Kansai Medical University Hospital, 2013‐0224: Nagoya University Hospital). An ethical problem assumed in this study was that the incidence of infectious complications in the non‐effective group might be increased, and that prolonged antibiotic prophylaxis might be associated with increased acquired antimicrobial resistance.

### 2.2 Randomization and antimicrobial prophylaxis dosage procedures

Patients were randomly assigned to either the 1‐day group or the 5‐day group before surgery. A central randomization system at Kansai Medical University was applied, and patients were stratified by institution.

In all patients, 1 g CZOP was given i.v. for 30 minutes immediately after induction, and additional doses were given once every 3 hours during the operative procedure. In addition, 1 g postoperative CZOP was given on the day of surgery for patients in the 1‐day group, whereas further doses of the postoperative CZOP were given every 12 hours for another 4 consecutive days in the 5‐day group (additional 2 g CZOP per day for 4 days).

### 2.3 Study endpoints

Primary endpoint was the incidence of postoperative infectious complications, which were defined as clinically relevant postoperative pancreatic fistula (CR‐POPF) which was defined according to International Study Group (ISGPF) criteria,^17^ intra‐abdominal abscess, postoperative cholangitis and wound infection. Secondary endpoints were overall postoperative morbidity, mortality, Clavien‐Dindo grade III~V complications,^18^ and length of postoperative hospital stay.

We diagnosed intra‐abdominal abscess by computed tomography (CT) or ultrasonography (US). If the drainage tube was already removed from the patient when we diagnosed the intra‐abdominal abscess, we inserted a percutaneous drainage tube. If the patient had a drainage tube in place, we exchanged it for a new tube because the tubes are vulnerable to obstruction by plugs consisting of fibrin and other materials. We investigated the drainage fluid for microbiological testing from the patients who were given a percutaneous drainage tube or who had been placed with a drainage tube for a long period. If the intra‐abdominal abscess was located in the center of the body where puncture was considered hazardous, we gave remedial antibiotics before considering surgical drainage.

### 2.4 Data collection

Clinical data were collected prospectively for all patients and included patient demographics, pathological examination, perioperative clinical information, and complications.

### 2.5 Statistical analysis

This study was designed to investigate that the infectious complication rate of the 1‐day group was not inferior to that of the 5‐day group. Based on previous studies,^18^, ^19^ the percentage of patients who developed infectious complications was expected to be 20%, and the threshold value was set at 40%. We planned to enrol at least 40 patients in each group without an accurate sample size calculation as a preparatory study.

Patient characteristics and perioperative and postoperative factors between the two groups were compared with Fisher's exact test and the Mann‐Whitney *U* test as appropriate. Statistical significance was defined as *P* < 0.05. Statistical analyses were carried out with the JMP statistical program version 13 (SAS Inc., Cary, NC, USA).

## 3 RESULTS

**Figure 2:**
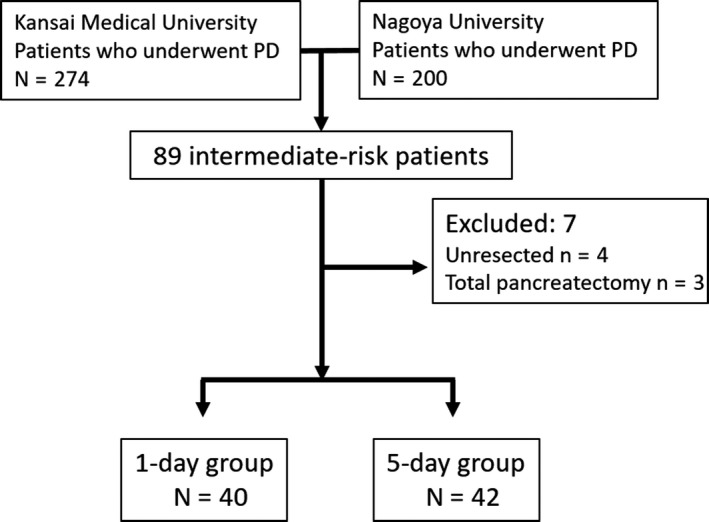
CONSORT diagram: Trial profile and patient allocation from March 2012 to December 2016. PD, pancreaticoduodenectomy

Between March 2012 and December 2016, 89 patients from the two institutions were pre‐registered as shown in the CONSORT diagram (Figure 2). Of these, a total of seven patients were excluded because of unresectable tumor (n = 4) and conversion to total pancreatectomy (n = 3). A total of 82 patients who underwent PD were randomized into the 1‐day group (n = 40) and the 5‐day group (n = 42).

### 3.1 Clinical characteristics

Patient characteristics are summarized in Table 1. Although most of the clinical background characteristics did not differ between groups, body mass index (BMI) in the 5‐day group (22.51 [15.90‐28.75]) was significantly higher than that in the 1‐day group (20.82 [14.88‐25.09], *P* = 0.012). The two types of fistula risk score proposed by Miller et al^19^ and Roberts et al^20^ were almost the same between groups. Neoadjuvant therapy was carried out in seven patients (17%) in the 1‐day group and in 10 patients (24%) in the 5‐day group. The majority of patients in both groups (85%) had a plastic stent inserted, whereas a metal stent had been the choice for the remaining 15%, with no significant difference in preference between groups. Median time from PBD to PD was 29 days in the 1‐day group and 38 days in the 5‐day group, which was not statistically significant.

**Table 1:** Patient characteristics and surgical factors

Patient characteristics	1‐day group (n = 40)	5‐day group (n = 42)	*P*‐value
Age, median (range), y	70 (47‐86)	72 (38‐84)	0.277
Gender, male: female, n (%)	22 (55) : 18 (45)	26 (62) : 16 (38)	0.526
Body mass index (range), kg/m^2^	20.82 (14.88‐25.09)	22.51 (15.90‐28.75)	0.012
Total‐bilirubin, median (range), mg/dL	0.8 (0.3‐6.5)	0.7 (0.4‐5.1)	0.544
C‐reactive protein, median (range), mg/dL	0.13 (0.0‐5.9)	0.14 (0.0‐2.1)	0.600
Creatinine, median (range), mg/dL	0.71 (0.46‐1.01)	0.72 (0.48‐1.23)	0.911
Pathological diagnosis, n (%)			0.300
Pancreatic ductal adenocarcinoma	17 (43)	20 (48)	0.541
Bile duct cancer	13 (32)	13 (31)	0.926
Peripapillary carcinoma	7 (18)	8 (19)	0.856
Other	3 (7)	1 (2)	0.273
Comorbid disease, − : +, n (%)	16 (40) : 24 (60)	19 (45) : 23 (55)	0.632
Diabetes mellitus, − : +, n (%)	32 (80) : 8 (20)	33 (79) : 9 (21)	0.873
Neo‐adjuvant therapy, − : +, n (%)	33 (83) : 7 (17)	32 (76) : 10 (24)	0.480
Type of preoperative biliary drainage; n (%)			
Plastic stent : metallic stent : CJ	34 (85) : 5 (13) : 1 (2)	36 (86) : 6 (14) : 0 (0)	0.400
Duration of preoperative biliary drainage, median (range), d	29 (3‐131)	38 (11‐372)	0.125
Surgical factors			
Operation time, median (range), min	425 (295‐575)	386 (305‐555)	0.121
Extent of blood loss median (range), mL	821 (133‐2750)	906 (273‐2754)	0.856
Type of pancreatojejunostomy, Kakita : Blumgart, n (%)	12 (30) : 28 (70)	16 (38) : 26 (62)	0.439
External stenting : Internal : None, n (%)	2 (5) : 8 (20) : 30 (75)	2 (5) : 12 (29) : 28 (66)	0.662
Main pancreatic duct, median (range), mm	4.0 (1‐15)	3.3 (1‐8)	0.554
Main pancreatic duct ≤3 mm, n (%)	18 (45)	21 (50)	0.650
Pancreatic parenchyma, soft : hard, n (%)	18 (45) : 22 (55)	25 (60) : 17 (40)	0.187
Fistula risk score,^19^ median (range)	4.5 (1‐10)	5.0 (1‐10)	0.378
Fistula risk score,^19^ Negligible : Low : Moderate : High, n (%)	0 : 8 (20) : 22 (55) : 10 (25)	0 : 6 (14) : 21 (50) : 15 (36)	0.531
Risk score,^20^ median (range)	8.34 (0.06‐25.24)	10.32 (1.37‐24.26)	0.180

CJ, cholecystojejunostomy; External, external stent; Internal, internal stent.

Kakita, modified Kakita method.^21^

Blumgart, modified Blumgart method.^22^

### 3.2 Surgical characteristics

Details on surgical parameters are also shown in Table 1. There were no significant differences in operation time and blood loss between the groups. Diameter of the main pancreatic duct and thickness of the pancreatic parenchyma were also similar. Additionally, the two groups were well matched for reconstruction method of pancreaticojejunostomy (PJ, modified Kakita method^21^ or modified Blumgart method^22^) and stent placement at PJ (external stent or internal stent or no stent).

### 3.3 Postoperative complications

Table 2 shows the postoperative complications in both groups. Incidence of overall infectious complications, including CR‐POPF, intra‐abdominal abscess, cholangitis and wound infection, was significantly lower in the 1‐day group compared with the 5‐day group (15% vs 36%, respectively; *P* = 0.029). Of the four types of complications, there were significant differences in the incidence of intra‐abdominal abscess (3% vs 21%, respectively; *P* = 0.005) and CR‐POPF (8% vs 24%, respectively; *P* = 0.038), but no difference in the incidence of postoperative cholangitis or wound infection.

**Table 2:** Comparison of postoperative complications and clinical outcomes

Postoperative complications	1‐day group (n = 40)	5‐day group (n = 42)	*P*‐value
Infectious complications, n (%)	**6 (15)**	**15 (36)**	**0.029**
Intra‐abdominal abscess, n (%)	**1 (3)**	**9 (21)**	**0.005**
Postoperative cholangitis, n (%)	**2 (5)**	**1 (2)**	**0.525**
Wound infection, n (%)	**1 (3)**	**1 (2)**	**0.972**
Clinically relevant POPF, n (%)	**3 (8)**	**10 (24)**	**0.038**
SIRS or sepsis, n (%)	6 (15)	11 (26)	0.208
Post‐pancreatectomy hemorrhage, n (%)	0 (0)	1 (2)	0.245
Delayed gastric emptying, n (%)	2 (5)	5 (12)	0.255
Overall POPF, n (%)	14 (35)	25 (60)	0.025
Overall complications, n (%)	22 (55)	32 (76)	0.042
Clavien‐Dindo classification ≥III, n (%)	4 (10)	13 (31)	0.017
Mortality, n (%)	0 (0)	0 (0)	1.000
Clinical outcomes			
Drain fluid AMY level in POD1, median range, IU/L	773 (20‐22 005)	2639 (25‐50 635)	0.032
Drain fluid AMY level in POD1 >4000 IU/L, n (%)	8 (20)	14 (33)	0.171
Duration of drainage tube placement, median (range), d	3 (2‐25)	3 (3‐31)	0.185
Percutaneous drainage, n (%)	2 (5)	5 (12)	0.256
Reoperation, n (%)	0 (0)	0 (0)	1.000
Use of remedial antibiotics, n (%)	11 (28)	18 (43)	0.144
1st‐, 2nd‐generation cephem, n	0	0	
3rd‐, 4th‐generation cephem, n	5	7	
Penicillin‐based antibiotics, n	1	4	
Carbapenem, n	1	3	
New quinolone, n	1	0	
Oral antimicrobial agents, n	3	4	
Postoperative date of initiated remedial antibiotics, median (range), d	8 (1‐14)	11 (5‐19)	0.125
Duration of in‐hospital stay, median (range), d	10 (8‐33)	15 (8‐44)	0.018

AMY, amylase; POD, postoperative day; POPF, postoperative pancreatic fistula defined by International Study Group (ISGPF); SIRS, systemic inflammatory response syndrome.

Infectious complications include clinically relevant POPF, intra‐abdominal abscess, postoperative cholangitis and wound infection.

Bold denotes primary endpoint.

Patients who had CR‐POPF had required percutaneous drainage, exchange of drainage tube, or remedial antibiotic. In the 1‐day group, three patients had CR‐POPF. Of these, percutaneous drainage was conducted in two patients and exchange of drainage tube in one patient, accompanied by giving of remedial antibiotics. In the 5‐day group, 10 patients had CR‐POPF. Of these, percutaneous drainage was conducted in five patients, exchange of drainage tube in four patients and persistent drainage with remedial antibiotics was needed in one patient. The incidence of POPF of all grades and overall morbidity were significantly lower in the 1‐day group compared with the 5‐day group (*P* < 0.05). In addition, the severe complication rate (Clavien‐Dindo III to V) was significantly lower in the 1‐day group compared with the 5‐day group (10% vs 31%, respectively; *P* = 0.017). Allergic reaction was not found in any patient who participated in this study.

### 3.4 Postoperative outcomes

As shown in Table 2, method of drain management did not differ significantly between groups, and drains were typically removed by postoperative day 3. Percutaneous drainage after removal was required in two patients (5%) in the 1‐day group and in five patients (12%) in the 5‐day group, which did not reach statistical significance. No reoperation was carried out in either of the groups, and there was no mortality. Remedial antimicrobials were initiated on postoperative day 8 as a median value in 11 patients (28%) of the 1‐day group and on postoperative day 11 in 18 patients (43%) of the 5‐day group for treatment of infectious complications and preventing aggravation of POPF, respectively. Postoperative hospital stay was significantly shorter in the 1‐day group compared with the 5‐day group (10 days vs 15 days, respectively; *P* = 0.018).

### 3.5 Microorganisms detected by abdominal drainage fluid culture

Potentially pathogenic microorganisms were identified from the intra‐abdominal drainage fluids of three patients with intra‐abdominal abscess or clinically relevant POPF in the 1‐day group and in 13 patients in the 5‐day group (Table 3). The most commonly isolated microorganisms were *Enterococcus* species and *Enterobacter* species. Anaerobic bacteria and methicillin‐resistant (MR) cocci were isolated exclusively from the drainage fluid of the 5‐day group.

**Table 3:** Microorganisms isolated from cultures derived from the intra‐abdominal drains

	1‐day group (n = 40)	5‐day group (n = 42)
Gram‐positive bacilli		
* Enterococcus* species		
*Enterococcus faecalis*	1	8
*Enterococcus faecium*	/	3
*Enterococcus avium*	/	1
Cocci (SA, SE, CNS, α*‐*streptococci)	/	2
Methicillin‐resistant cocci (SA, SE, CNS)	/	4
Gram‐negative bacilli		
*Enterobacter* species		
*Enterobacter cloacae*	/	2
*Enterobacter aerogenes*	/	1
*Klebsiella pneumoniae*	2	1
*Citrobacter braakii*	1	/
*Serratia plymuthica*	1	1
Anaerobic bacteria		
*Fusobacterium necrophorum*	/	1
*Prevotella bivia*	/	1

CNS, coagulase‐negative staphylococci; SA, *Staphylococcus aureus*; SE, *Staphylococcus epidermidis*; /, no detection.

### 3.6 Risk factors for infectious complications

Risk factors for infectious complications are shown in Table 4. Univariate analyses by original parameters showed that non‐pancreatic disease, soft pancreatic parenchyma and the 5‐day group were significantly associated with infectious complications. BMI was found not to be a risk factor for infectious complications in this analysis. Multivariate analysis was performed using factors with significant differences in univariate analyses. Duration of CZOP dosage (5 days) was only independent risk factor for infectious complications.

**Table 4:** Risk factors for infectious complications

Parameter	Univariate analysis	*P*‐value	Multivariate analysis	*P*‐value
	OR (95% CI)		OR (95% CI)	
Age, years	1.47 (0.54‐4.09)	0.447		
Body mass index	1.20 (0.67‐1.00)	0.052		
Comorbid disease, − : +	0.88 (0.32‐2.48)	0.810		
Diabetes mellitus, − : +	0.61 (0.13‐2.14)	0.470		
Disease, panc, non‐panc	2.76 (0.98‐8.61)	0.049	2.36 (0.47‐12.59)	0.294
Neoadjuvant therapy, − : +	0.61 (0.13‐2.14)	0.470		
Serum albumin level, g/dL	1.49 (0.29‐2.10)	0.496		
Operation time, min	1.00 (0.98‐1.00)	0.366		
PJ anastomosis, Kakita vs Blumgart	1.65 (0.58‐4.60)	0.334		
Main pancreatic duct width, mm	1.25 (0.95‐1.73)	0.119		
Pancreatic parenchyma, soft vs hard	2.94 (1.05‐9.21)	0.040	1.12 (0.28‐7.34)	0.672
Drain fluid AMY level in POD1, ≤4000 vs >4000 IU/L	2.77 (0.95‐8.06)	0.062		
Duration of CZOP dosage, days 1 vs 5	0.31 (0.10‐0.89)	0.029	0.31 (0.09‐0.93)	0.034

AMY, amylase; CI, confidence interval; CZOP, cefozopran; OR, odds ratio; panc, pancreatic disease; PJ, pancreaticojejunostomy; POD, postoperative day.

## 4 DISCUSSION

Current guidelines recommend discontinuation of antimicrobial prophylaxis within 24 hours, even after major hepatobiliary and pancreatic surgery.^11^, ^12^ First‐ or second‐generation cephalosporins as prophylactic antimicrobial therapy on the day of surgery only could indeed have been appropriate for patients who underwent PD without PBD, which comprised 41% of the patients who underwent PD during the study period and who were not eligible for the present study. These patients had been categorized as the low‐risk group for postoperative infection. In contrast, patients who developed cholangitis before PD (the high‐risk group, amounting to 16% of patients who underwent PD during the study period) may require third‐ or fourth‐generation cephalosporins until postoperative day 4. However, there has been no consensus on the optimal duration of antimicrobial prophylaxis in patients with PBD and without cholangitis (35% of patients who underwent PD) which, in our system, had been classified as the intermediate‐risk group. The current RCT indicated that 1‐day administration of antimicrobial prophylaxis with CZOP is sufficient for this group of patients.

Recently, several RCT which assessed the duration of antimicrobial prophylaxis in other types of surgery have been reported. In gastric surgery, it was reported that elimination of postoperative antimicrobial prophylaxis did not increase the incidence of surgical‐site infections.^23^ Furthermore, 2‐day administration of antimicrobial prophylaxis is sufficient for patients undergoing hepatectomy with extrahepatic bile duct resection, which is one of the most invasive types of surgery, compared with 4‐day administration.^24^ This study was intended as a preparatory study designed to show that the infectious complication rate of the 1‐day group was not inferior to that of the 5‐day group. Unexpectedly, giving postoperative antimicrobial prophylaxis for 5 days after PD was shown to significantly increase the incidence of infectious complications compared with the 1‐day dosage strategy. Consequently, the 5‐day group was associated with prolonged duration of in‐hospital stay.

Infectious complications including CR‐POPF after PD result in a prolonged and complex postoperative course that is sometimes associated with mortality.^3^ This could lead to serious oncological consequences, especially for patients with pancreatic ductal carcinoma, as a prolonged time to recovery may delay the initiation of evidence‐based adjuvant chemotherapy. Postoperative complications have been documented to adversely affect long‐term survival in several cancer types.

Preoperative biliary drainage before PD may not always be indicated for patients with obstructive jaundice, provided the patient does not suffer from cholangitis. However, in clinical practice, patients often receive PBD at local hospitals before being given the final diagnosis, after which they are sent to high‐volume centers for surgery. Furthermore, neoadjuvant chemo(radio)therapy has recently been indicated for increasing numbers of patients with pancreatic ductal adenocarcinoma. For these patients, restoration of adequate liver function through PBD is mandatory, even if the drainage may in itself be a risk factor for biliary contamination and postoperative infectious complications.^5^, ^8^, ^24^ Thus, PBD remains necessary, at least in some patients for whom major pancreatic surgery will eventually be indicated. Consequently, the time lapse between the PBD and PD tended to be prolonged in this report as compared with the high‐volume US centers.

Previous studies showed that bile juice was contaminated with bacteria in more than 80% of patients who underwent PBD.^4^, ^25^ Biliary tract infection and intestinal colonized bile juice can lead to contamination of the peritoneal cavity during biliary duct transection or hepaticojejunostomy. To reduce infectious complications after PD, appropriate antimicrobial prophylaxis targeting intrabiliary microorganisms should be given. The most commonly isolated microorganisms from bile cultures were *Enterococcus* species, *Enterobacter* species and *Klebsiella* species,^4^, ^26^ consistent with our findings (Table 3). Additionally, two previous reports that evaluated bacteria from ascitic fluid after PD reported that most of the isolated bacteria were *Enterococcus* and *Enterobacter* species.^27^, ^28^ Thus, broad‐spectrum antimicrobials targeting these microorganisms are recommended for patients who will undergo PD following PBD. In the current RCT, we used CZOP as antimicrobial prophylaxis covering *Enterococcus, Enterobacter* and *Klebsiella* species.

In the 1‐day group, which had better clinical outcomes, the incidence of overall infectious complications was 15% and that of intra‐abdominal infections was only 8%, which were lower than those reported in previous studies.^29^, ^30^ An important finding from this study was that anaerobic bacteria and MR cocci including *Staphylococcus aureus* and *Staphylococcus epidermis* were isolated exclusively from the 5‐day group, and not from the 1‐day group. These results imply that CZOP might be an appropriate choice as antimicrobial prophylaxis in limited patients (middle‐ or high‐risk groups who undergo PD), provided that prolonged usage in the absence of clear signs of infection is avoided.

Harmful effects of prolonged prophylactic use of antimicrobial agents have been documented after other types of major surgery. Anaerobic bacteria were reportedly isolated from patients who developed postoperative infections following radical cystectomy and urinary diversion using the small intestine, when cephalosporin was continued for 1 week as antimicrobial prophylaxis.^31^ In cardiac surgery, prolonged prophylaxis with antimicrobials has been associated with an increased risk of acquired antimicrobial resistance.^32^ Moreover, it is well known that long‐term postoperative antimicrobial prophylaxis has led to outbreaks of postoperative methicillin‐resistant *Staphylococcus aureus* (MRSA) infection.^33^ In addition, the incidence of patients with signs of infection in whom causative pathogens cannot be detected by conventional methodology has been increasing. Recently, an analysis using the 16s ribosomal RNA gene showed higher sensitivity in detecting anaerobic bacteria,^34^ but, at the same time, suggested the possibility that the consequences of inadequate prophylaxis antimicrobials are more serious than they seem.

Development of CR‐POPF is the most important risk factor for intra‐abdominal infections.^35^ Pancreatic surgeons seek to develop techniques for reducing the incidence of CR‐POPF, including various methods of PJ anastomosis, pancreatic duct stenting,^36^ and drain management.^37^ In addition, several risk factors for the development of CR‐POPF, including soft parenchyma of the pancreas,^38^ a non‐dilated pancreatic duct,^39^ drain fluid amylase level >4000 or 5000 IU/L at postoperative day 1 (POD1)^40^, ^41^ and fistula risk score^19^, ^20^ have been identified to construct strategies to prepare for some inevitable complications. In the current study, the incidence of CR‐POPF in the 5‐day group was significantly higher, although there were no marked differences in pancreatic texture and duct diameter, surgical methods, duration of drainage tube placement, rate of patients with drain fluid amylase level >4000 IU on POD1 and two types of fistula risk score^19^, ^20^ between the groups. However, the value of BMI and drain fluid amylase level at POD1 in the 5‐day group were significantly higher compared with the 1‐day group by univariate analysis. These factors might influence the development of CR‐POPF. However, BMI and drain fluid amylase level at POD1 >4000 IU/L were not independent risk factors for the incidence of CR‐POPF by multivariate analysis (data not shown). The most important finding of the current study was that infectious complications were not increased by limited use of antimicrobial as shown in Table 2. These findings indicate that the prolonged use of antimicrobials could mar all of the painstaking efforts of the surgical team to reduce the morbidity and mortality associated with pancreatic surgery.

In the current study, the two participating institutes carried out mutual site visits and had taken several steps to standardize the surgical technique and perioperative management, with particular focus on the evaluation of CR‐POPF and placement, removal and exchange of the drainage tubes. Our result that the short administration of antibiotics results in superior outcomes in PD was unexpected, but is actually true in other less complex surgical procedures. Perhaps an optimal duration of antibiotics does not solely depend on the complexity of surgery. However, a larger study with a greater number of participating institutes will be needed to confirm our findings.

We are aware of some limitations of this study. First, this was a randomized but unblinded study with a small sample size. The current study was intended as a preparatory study designed to explore whether the issue of the duration of prophylaxis antimicrobials needs further and more extensive exploration in larger studies. Second, there was a significant difference in the baseline BMI of patients between the two groups. Obesity was admittedly one of the risk factors for clinically relevant POPF and postoperative infectious complications. Stratification by BMI was not carried out in this study because, again, the number of enrolled patients was too small for such considerations. In addition, although differences in the incidence of other potential risk factors for POPF, such as pancreatic texture, surgical procedure, fistula risk score and drain management were not statistically significant, one could not completely deny the possibility that some differences in background characteristics could have biased our results.

In conclusion, 1‐day administration of broad‐spectrum antimicrobial prophylaxis is appropriate as antimicrobial prophylaxis in patients who undergo PD after PBD and are without preoperative cholangitis, and long‐term administration of antimicrobial prophylaxis in the absence of apparent signs of infection should be avoided.

## 5 DISCLOSURE

Conflicts of Interest: Authors declare no conflicts of interest for this article.

Author Contribution: Sohei Satoi, Tomohisa Yamamoto and Masaya Kotsuka contributed to all aspects of this study and article. Tsutomu Fujii, Suguru Yamada, Hiroaki Yanagimoto, So Yamaki, Hideki Takami, and Takayuki Miyara contributed to study conception and design, collection of the data and critical revision of the article. Satoshi Hirooka, Hisashi Kosaka, Masaya Kotsuka, and Yasuhiro Kodera contributed to collection of the data and critical revision of the article. All authors approved the final draft of the article.

## References

[1] Vin Y , Sima CS , Getrajdman GI , et al. Management and outcomes of postpancreatectomy fistula, leak, and abscess: results of 908 patients resected at a single institution between 2000 and 2005. J Am Coll Surg. 2008;207:490–8. 18926450 10.1016/j.jamcollsurg.2008.05.003

[2] Yeo CJ , Cameron JL , Sohn TA , et al. Six hundred fifty consecutive pancreaticoduodenectomies in the 1990s: pathology, complications, and outcomes. Ann Surg. 1997;226:248–57; discussion 57‐60. 9339931 10.1097/00000658-199709000-00004 PMC1191017

[3] Kimura W , Miyata H , Gotoh M , et al. A pancreaticoduodenectomy risk model derived from 8575 cases from a national single‐race population (Japanese) using a web‐based data entry system: the 30‐day and in‐hospital mortality rates for pancreaticoduodenectomy. Ann Surg. 2014;259:773–80. 24253151 10.1097/SLA.0000000000000263

[4] Sudo T , Murakami Y , Uemura K , et al. Specific antibiotic prophylaxis based on bile cultures is required to prevent postoperative infectious complications in pancreatoduodenectomy patients who have undergone preoperative biliary drainage. World J Surg. 2007;31:2230–5. 17726628 10.1007/s00268-007-9210-4

[5] Cortes A , Sauvanet A , Bert F , et al. Effect of bile contamination on immediate outcomes after pancreaticoduodenectomy for tumor. J Am Coll Surg. 2006;202:93–9. 16377502 10.1016/j.jamcollsurg.2005.09.006

[6] van der Gaag NA , Kloek JJ , de Castro SM , Busch OR , van Gulik TM , Gouma DJ . Preoperative biliary drainage in patients with obstructive jaundice: history and current status. J Gastrointest Surg. 2009;13:814–20. 18726134 10.1007/s11605-008-0618-4

[7] Moole H , Bechtold M , Puli SR . Efficacy of preoperative biliary drainage in malignant obstructive jaundice: a meta‐analysis and systematic review. World J Surg Oncol. 2016;14:182. 27400651 10.1186/s12957-016-0933-2 PMC4940848

[8] van der Gaag NA , Rauws EA , van Eijck CH , et al. Preoperative biliary drainage for cancer of the head of the pancreas. N Engl J Med. 2010;362:129–37. 20071702 10.1056/NEJMoa0903230

[9] Sumiyama Y , Kusachi S , Yoshida Y , et al. Questionnaire on perioperative antibiotic therapy in 2003: postoperative prophylaxis. Surg Today. 2006;36:107–13. 16440154 10.1007/s00595-005-3112-6

[10] Mezhir JJ , Brennan MF , Baser RE , et al. A matched case‐control study of preoperative biliary drainage in patients with pancreatic adenocarcinoma: routine drainage is not justified. J Gastrointest Surg. 2009;13:2163–9. 19774424 10.1007/s11605-009-1046-9

[11] Mangram AJ , Horan TC , Pearson ML , Silver LC , Jarvis WR . Guideline for prevention of surgical site infection, 1999. Centers for disease control and prevention (CDC) hospital infection control practices advisory committee. Am J Infect Control. 1999;27:97–132; quiz 3‐4; discussion 96. 10196487

[12] Bratzler DW , Dellinger EP , Olsen KM , et al. Clinical practice guidelines for antimicrobial prophylaxis in surgery. Surg Infect (Larchmt). 2013;14:73–156. 23461695 10.1089/sur.2013.9999

[13] Sourrouille I , Gaujoux S , Lacave G , et al. Five days of postoperative antimicrobial therapy decreases infectious complications following pancreaticoduodenectomy in patients at risk for bile contamination. HPB (Oxford). 2013;15:473–80. 23458261 10.1111/hpb.12012 PMC3664052

[14] Wada K , Takada T , Kawarada Y , et al. Diagnostic criteria and severity assessment of acute cholangitis: Tokyo Guidelines. J Hepatobiliary Pancreat Surg. 2007;14:52–8. 17252297 10.1007/s00534-006-1156-7 PMC2784515

[15] Yanagimoto H , Satoi S , Yamamoto T , et al. Clinical impact of preoperative cholangitis after biliary drainage in patients who undergo pancreaticoduodenectomy on postoperative pancreatic fistula. Am Surg. 2014;80:36–42. 24401513

[16] Kitahata Y , Kawai M , Tani M , et al. Preoperative cholangitis during biliary drainage increases the incidence of postoperative severe complications after pancreaticoduodenectomy. Am J Surg. 2014;208:1–10. 24530042 10.1016/j.amjsurg.2013.10.021

[17] Bassi C , Marchegiani G , Dervenis C , et al. The 2016 update of the International Study Group (ISGPS) definition and grading of postoperative pancreatic fistula: 11 years after. Surgery. 2017;161:584–91. 28040257 10.1016/j.surg.2016.11.014

[18] Clavien PA , Barkun J , de Oliveira ML , et al. The Clavien‐Dindo classification of surgical complications: five‐year experience. Ann Surg. 2009;250:187–96. 19638912 10.1097/SLA.0b013e3181b13ca2

[19] Miller BC , Christein JD , Behrman SW , et al. A multi‐institutional external validation of the fistula risk score for pancreatoduodenectomy. J Gastrointest Surg. 2014;18:172–9; discussion 9‐80. 24002771 10.1007/s11605-013-2337-8

[20] Roberts KJ , Sutcliffe RP , Marudanayagam R , et al. Scoring system to predict pancreatic fistula after pancreaticoduodenectomy: a UK multicenter study. Ann Surg. 2015;261:1191–7. 25371115 10.1097/SLA.0000000000000997

[21] Satoi S , Toyokawa H , Yanagimoto H , et al. Is a nonstented duct‐to‐mucosa anastomosis using the modified Kakita method a safe procedure? Pancreas. 2010;39:165–70. 19959970 10.1097/MPA.0b013e3181bd672c

[22] Fujii T , Sugimoto H , Yamada S , et al. Modified Blumgart anastomosis for pancreaticojejunostomy: technical improvement in matched historical control study. J Gastrointest Surg. 2014;18:1108–15. 24733259 10.1007/s11605-014-2523-3

[23] Imamura H , Kurokawa Y , Tsujinaka T , et al. Intraoperative versus extended antimicrobial prophylaxis after gastric cancer surgery: a phase 3, open‐label, randomised controlled, non‐inferiority trial. Lancet Infect Dis. 2012;12:381–7. 22297080 10.1016/S1473-3099(11)70370-X

[24] Sugawara G , Yokoyama Y , Ebata T , et al. Duration of antimicrobial prophylaxis in patients undergoing major hepatectomy with extrahepatic bile duct resection: a randomized controlled trial. Ann Surg. 2018;267:142–8. 27759623 10.1097/SLA.0000000000002049

[25] Howard TJ , Yu J , Greene RB , et al. Influence of bactibilia after preoperative biliary stenting on postoperative infectious complications. J Gastrointest Surg. 2006;10:523–31. 16627218 10.1016/j.gassur.2005.08.011

[26] Fong ZV , McMillan MT , Marchegiani G , et al. Discordance between perioperative antibiotic prophylaxis and wound infection cultures in patients undergoing pancreaticoduodenectomy. JAMA Surg. 2016;151:432–9. 26720272 10.1001/jamasurg.2015.4510

[27] Sugiura T , Mizuno T , Okamura Y , et al. Impact of bacterial contamination of the abdominal cavity during pancreaticoduodenectomy on surgical‐site infection. Br J Surg. 2015;102:1561–6. 26206386 10.1002/bjs.9899

[28] Nagakawa Y , Matsudo T , Hijikata Y , et al. Bacterial contamination in ascitic fluid is associated with the development of clinically relevant pancreatic fistula after pancreatoduodenectomy. Pancreas. 2013;42:701–6. 23429497 10.1097/MPA.0b013e31826d3a41

[29] Okano K , Hirao T , Unno M , et al. Postoperative infectious complications after pancreatic resection. Br J Surg. 2015;102:1551–60. 26387569 10.1002/bjs.9919

[30] Sudo T , Murakami Y , Uemura K , et al. Perioperative antibiotics covering bile contamination prevent abdominal infectious complications after pancreatoduodenectomy in patients with preoperative biliary drainage. World J Surg. 2014;38:2952–9. 25022981 10.1007/s00268-014-2688-7

[31] Hiyama Y , Takahashi S , Uehara T , et al. Significance of anaerobic bacteria in postoperative infection after radical cystectomy and urinary diversion or reconstruction. J Infect Chemother. 2013;19:867–70. 23504391 10.1007/s10156-013-0583-z

[32] Harbarth S , Samore MH , Lichtenberg D , Carmeli Y . Prolonged antibiotic prophylaxis after cardiovascular surgery and its effect on surgical site infections and antimicrobial resistance. Circulation. 2000;101:2916–21. 10869263 10.1161/01.cir.101.25.2916

[33] Fukatsu K , Saito H , Matsuda T , Ikeda S , Furukawa S , Muto T . Influences of type and duration of antimicrobial prophylaxis on an outbreak of methicillin‐resistant Staphylococcus aureus and on the incidence of wound infection. Arch Surg. 1997;132:1320–5. 9403537 10.1001/archsurg.1997.01430360066012

[34] Kawanami T , Fukuda K , Yatera K , Kido M , Mukae H , Taniguchi H . A higher significance of anaerobes: the clone library analysis of bacterial pleurisy. Chest. 2011;139:600–8. 20688923 10.1378/chest.10-0460

[35] Kanda M , Fujii T , Kodera Y , Nagai S , Takeda S , Nakao A . Nutritional predictors of postoperative outcome in pancreatic cancer. Br J Surg. 2011;98:268–74. 20960457 10.1002/bjs.7305

[36] Motoi F , Egawa S , Rikiyama T , Katayose Y , Unno M . Randomized clinical trial of external stent drainage of the pancreatic duct to reduce postoperative pancreatic fistula after pancreaticojejunostomy. Br J Surg. 2012;99:524–31. 22497024 10.1002/bjs.8654

[37] Bassi C , Molinari E , Malleo G , et al. Early versus late drain removal after standard pancreatic resections: results of a prospective randomized trial. Ann Surg. 2010;252:207–14. 20622661 10.1097/SLA.0b013e3181e61e88

[38] Poon RT , Fan ST , Lo CM , et al. External drainage of pancreatic duct with a stent to reduce leakage rate of pancreaticojejunostomy after pancreaticoduodenectomy: a prospective randomized trial. Ann Surg. 2007;246:425–33; discussion 33‐5. 17717446 10.1097/SLA.0b013e3181492c28 PMC1959348

[39] Yamamoto T , Satoi S , Yanagimoto H , et al. Clinical effect of pancreaticojejunostomy with a long‐internal stent during pancreaticoduodenectomy in patients with a main pancreatic duct of small diameter. Int J Surg. 2017;42:158–63. 28465259 10.1016/j.ijsu.2017.04.056

[40] Kawai M , Kondo S , Yamaue H , et al. Predictive risk factors for clinically relevant pancreatic fistula analyzed in 1,239 patients with pancreaticoduodenectomy: multicenter data collection as a project study of pancreatic surgery by the Japanese Society of Hepato‐Biliary‐Pancreatic Surgery. J Hepatobiliary Pancreat Sci. 2011;18:601–8. 21491103 10.1007/s00534-011-0373-x

[41] Molinari E , Bassi C , Salvia R , et al. Amylase value in drains after pancreatic resection as predictive factor of postoperative pancreatic fistula: results of a prospective study in 137 patients. Ann Surg. 2007;246:281–7. 17667507 10.1097/SLA.0b013e3180caa42f PMC1933557

